# Impact of natalizumab on patient-reported outcomes in multiple sclerosis: a longitudinal study

**DOI:** 10.1186/1477-7525-10-155

**Published:** 2012-12-27

**Authors:** Judith J Stephenson, David M Kern, Sonalee S Agarwal, Ruth Zeidman, Krithika Rajagopalan, Siddhesh A Kamat, John Foley

**Affiliations:** 1 HealthCore Inc., 800 Delaware Avenue, Fifth Floor, Wilmington, DE, 19801, USA; 2 Biogen Idec Inc., 14 Cambridge Center, Cambridge, MA, 02142, USA; 3 Medaxial Group, 61 Webber Street, London, SE1 0RF, UK; 4 Rocky Mountain Multiple Sclerosis Clinic, Rocky Mountain Neurological Associates, 370 E 9th Ave, STE 106, Salt Lake City, UT, 84103, USA

**Keywords:** Cognitive function, Health-related quality of life, Multiple sclerosis, Natalizumab, Patient-reported outcomes

## Abstract

**Background:**

Natalizumab (Tysabri, Biogen Idec and Elan Pharmaceuticals) significantly reduces the relapse rate and disability progression, and improves health-related quality of life (HRQoL), in patients with relapsing-remitting multiple sclerosis. We investigated the impact of natalizumab on patient-reported outcomes (PROs) in a real-world setting.

**Methods:**

PRO data were collected from patients enrolled in a longitudinal real-world study using validated measures administered as surveys before the patients initiated natalizumab treatment and after the 3rd, 6th, and 12th monthly infusion. HRQoL, ability to carry out daily activities, disability level, and impact on cognitive functioning and fatigue were assessed.

**Results:**

A total of 333 patients completed 12 months of assessments. After 12 months of natalizumab treatment, 69% to 88% of patients reported a positive outcome (either an improvement or no further decline) in all PRO measures assessed. Significant improvements in general and disease-specific HRQoL were observed after three infusions, both with physical (p < .01) and psychological (p < .001) measures, and were sustained after 12 infusions (all p < .001). The impact of multiple sclerosis on cognitive functioning and fatigue was significantly reduced (both p < .001 after 3 and 12 infusions).

**Conclusions:**

PRO measures were improved with natalizumab in a real-world setting. The improvements were observed as early as after 3 months and sustained over a 12-month period. The improvements in PROs show that, in clinical practice, the clinical benefits of natalizumab are translated into patient-reported benefits.

## Background

Multiple sclerosis (MS) patients commonly experience a range of debilitating symptoms [1]. The progressive nature of the disease leads to increasing disability, with both physical and mental impairment concomitant with increasing impact on patients’ general quality of life, family and social life, and employment status [2,3]. MS patients rank their quality of life to be lower than not only that of the general population but also lower than that of patients with other chronic diseases [4-6]. MS, which affects mainly young adults, imposes a substantial economic burden on society, reflected both in healthcare costs and loss of productivity [7]. In general, MS therapy aims to reduce the rate of disease relapses, delay disease progression, and manage the symptoms of the disease [1].

Natalizumab (Tysabri^a^, Biogen Idec and Elan Pharmaceuticals) is indicated as monotherapy for treatment of remitting-relapsing MS. In the AFFIRM and SENTINEL studies, natalizumab was demonstrated to significantly reduce annualized relapse rate and the risk of sustained disability and to reduce disease activity over 2 years [8-10]. In addition to clinical measures, improvements in health-related quality of life (HRQoL), which is a patient-reported outcome (PRO), were also reported [11]. A positive impact of natalizumab on PROs has also been demonstrated in short-term and small, observational, real-world studies [12,13].

Given the impact of MS on patient lives, it is important to complement clinical evidence with PRO evidence, as it provides insight into patients' condition from their own perspective [14]. PROs assessed in the context of real-world observational studies may more accurately reflect the effectiveness of a drug in the general patient population, as perceived by patients themselves, than in a clinical trial where certain patient groups may have been excluded.

The study reported here is the first large, real-world, 12-month longitudinal study assessing MS patients’ experiences with natalizumab in the United States, across a comprehensive range of PRO measures.

## Methods

### Study design and patients

**Figure 1:**
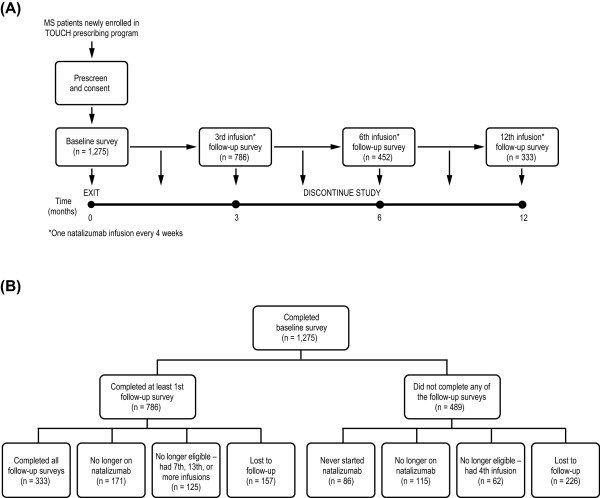
**(A) Study design.** (**B**) Disposition of patients during the 12-month real-world longitudinal study. *MS* multiple sclerosis.

A 1-year, longitudinal, observational, single-arm study was designed to assess MS patient experiences before and after starting treatment with natalizumab at a monthly dose of 300 mg administered intravenously (Figure 1A). The study consisted of four 20–25-minute surveys administered at baseline before initiating natalizumab, and three follow-up surveys administered between the 3rd and 4th, the 6th and 7th, and the 12th and 13th infusions.

Patients who were newly prescribed natalizumab in the United States were identified through the manufacturer’s restricted drug distribution program, the Tysabri Outreach Unified Commitment to Health (TOUCH^b^) [15], and were contacted between July 2008 and January 2009 by telephone by trained interviewers. The Human Subjects Committee of the New England Institutional Review Board approved the protocol, recruitment, consent, and survey procedures. All patient data were handled in compliance with the regulations of the US Insurance Portability and Accountability Act of 1996.

Study participation eligibility criteria were the ability to understand English, age greater than 18 years, the ability to provide informed verbal consent, and a prescription for natalizumab without the patient having received the first infusion. Potential participants were excluded if they had already had the 4th, 7th, or 13th infusion, respectively, at the time of the follow-up surveys, or if they were no longer receiving natalizumab after the baseline survey.

### PRO measures

Patients completed the following assessments: general and disease-specific HRQoL, ability to carry out daily activities, disability level, cognitive functioning, and fatigue status. The baseline survey also contained questions relating to patient demographics and clinical characteristics.

#### General HRQoL (Short-Form 12 Version 2 [SF-12v2])

The SF-12v2 is a generic HRQoL measure that consists of 12 questions selected from the widely used SF-36 health survey [16]. It was also used in the AFFIRM and SENTINEL clinical trials [11]. It provides information on physical and mental health via the Physical and Mental Component Summary (PCS and MCS) scores, which are calculated as norm-based scores ranging from 0 to 100. Higher scores represent better HRQoL, with the average US population score being 50 for each [17]. A score change of 5 points in MCS or PCS scales is considered to be clinically meaningful [18]. No change or an improvement of at least 5 points was assumed in this study to be a positive outcome for the patient.

#### Disease-specific HRQoL (Multiple Sclerosis Impact Scale [MSIS]-29)

The MSIS-29 is a disease-specific HRQoL measure. It assesses the physical (20 questions) and psychological (9 questions) impact of MS, provided as separate scores on a scale of 0 to 100, where lower scores represent better HRQoL [19]. MSIS-29 scores can be categorized so that 0–19 represent “no problems”; 20–39, “few problems”; 40–59, “moderate problems”; 60–79, “quite a few problems”; and 80–100, “extreme problems” [20]. No change or an improvement of at least one score category was assumed in this study to be a positive outcome for the patient.

#### Ability to perform daily activities (Functional Status [FS])

The FS scale assesses the ability of MS patients to perform their normal daily activities at the present time using a single question with a 5-point scale ranging from FS1 to FS5, with FS5 representing the greatest impairment [Additional file 1. The scale is modified from a self-assessment questionnaire measuring neurological impairment [21]. No change or an improvement of at least one step of the scale was assumed in this study to be a positive outcome for the patient.

#### Disability level (Disease Steps [DS])

The DS scale consists of a single item that assesses MS patients’ level of disability on a 7-point scale ranging from DS0 to DS6, with DS6 representing the greatest disability [22] [Additional file 2. It is validated against the Expanded Disability Status Scale [23], a widely used physician-reported measure of disability. No change or an improvement of least one step on the scale was assumed in this study to be a positive outcome for the patient.

#### Cognitive functioning (Medical Outcomes Scale-Cognitive Functioning [MOS-Cog])

The MOS-Cog instrument measures the frequency with which MS has an impact on cognitive function [24]. It consists of six items, with responses ranging from 1 (“All of the time”) to 6 (“None of the time”). Scores range from 6 to 36, with higher scores representing less impact on cognitive functioning. No change or an improvement of least one point on the scale was assumed in this study to be a positive outcome for the patient.

#### Fatigue status (Modified Fatigue Impact Scale [MFIS]-5)

The MFIS-5 is an abbreviated version of the MFIS-21, which is a modified form of the Fatigue Impact Scale [25,26]. The MFIS-5 consists of five questions that assess the impact of fatigue on physical, cognitive, and psychosocial functioning, with five response levels ranging from 0 (“Never”) to 4 (“Almost always”). Total scores range from 0 to 20, with higher scores representing a greater impact of fatigue. No change or an improvement of least one point on the scale was assumed in this study to be a positive outcome for the patient.

### Statistical methods and sensitivity analysis

Univariate analyses were performed to describe baseline demographics and compare PROs at each time point. Linear mixed effects models were used to evaluate outcome changes over time, controlling for age, disability level, functional status, years since diagnosis, number of comorbidities, and number of MS drugs used prior to natalizumab. Adjusted mean scores for all outcomes estimated from the linear mixed effects models are reported. Statistical Analysis System (SAS) version 9.2 was used for all analyses (SAS Institute Inc., Cary, NC, USA).

Two types of sensitivity analysis were performed to assess the validity of the results and to ensure that loss of patients during follow-up did not cause any bias in the results: (1) the baseline characteristics, patient-reported level of disability, and years since diagnosis of patients who completed all three follow-up surveys were compared with corresponding variables of patients who completed only the baseline survey; and (2) mixed-effects models were developed using all patients who had received at least one natalizumab infusion to test the robustness of the models.

## Results

### Patients

A total of 1,275 patients fulfilled the inclusion criteria and completed the baseline survey. At the end of the 12-month follow-up, 786 patients had completed at least one follow-up survey – 333 patients (42%) had completed all three follow-up surveys and 157 patients had withdrawn from the study. In total, 559 patients were excluded as they never started, were no longer taking natalizumab, or had received too many infusions at the time of the follow-up surveys. There were 226 patients who were lost to follow-up and did not complete any follow-up surveys (Figure 1B).

The study participants’ average age was 47 years (standard deviation 10 years). The majority were female and Caucasian, reflecting the MS patient population as a whole [1]. Almost all patients had been treated with one or more disease-modifying treatments (DMTs) before initiating natalizumab. Table 1 summarizes patient demographics and characteristics at baseline.

**Table 1:** Patient demographics and characteristics at baseline

Age, years; mean (SD)	46.8 (10.4)
Female (%)	78.1
Caucasian (%)	86.7
Years since MS diagnosis; mean (SD)	10.6 (7.9)
Number of MS DMTs used prior to natalizumab; mean (SD)	2.0 (1.1)
Proportion of patients treated with 1 DMT prior to natalizumab	35
Proportion of patients treated with ≥ 2 DMTs prior to natalizumab	62
SF-12v2 PCS; mean (SE)	34.2 (0.6)
SF-12v2 MCS; mean (SE)	43.2 (0.7)
Proportion of patients with SF-12v2 PCS ≤ 50^a^	91
Proportion of patients with SF-12v2 MCS ≤ 50^a^	69
FS; mean (SD)	2.6 (1.1)^b^
DS; mean (SD)	2.8 (1.8)^c^

There was considerable impact of MS on patients’ lives at baseline. The majority of patients reported general HRQoL scores below those of the US general population. Additionally, 86% reported being at FS2 or above, meaning that their ability to perform daily activities was impaired, ranging from mild limitations to requiring assistance with basic self-care. Almost all patients (96%) also reported being at DS1 or above, meaning that they experienced some degree of disability.

### General and disease-specific HRQoL

**Figure 2:**
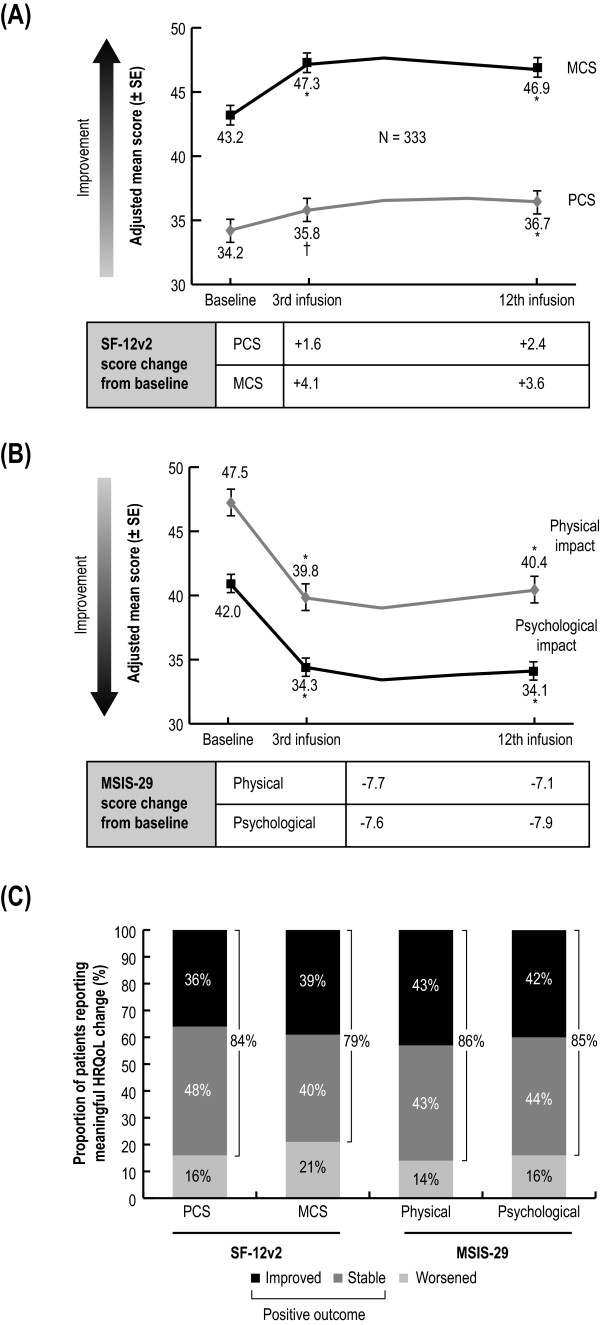
**Changes in general and disease-specific HRQoL over 12 natalizumab infusions.** (**A**) Mean SF-12v2 scores (± SE). (**B**) MSIS-29 scores (± SE) at baseline prior to natalizumab initiation and after the 3rd and 12th infusion. Because of missing data for covariates in their respective models, n values for SF-12v2 were 317 for PCS and 320 for MCS, and 328 for MSIS-29. p values were from analyses of variance performed on the adjusted means estimated by the mixed-effect model controlling for infusion, age, baseline DS, baseline FS, and also for years since diagnosis for the PCS/physical analyses. (**C**) Proportion of patients with improved, stable, or worsened HRQoL. The scores had to change by at least 5 points for SF-12v2, or by 19 points (one score interpretation category for MSIS-29) for the patient’s HRQoL to be considered improved or worsened. *p < .0001; ^†^p < .01. *HRQoL* health-related quality of life; *MCS* Mental Component Summary;* MSIS-29* Multiple Sclerosis Impact Scale 29; *PCS* Physical Component Summary; *SE* standard error; *SF-12v2* Short-Form 12 Version 2.

Patients reported statistically significant improvements in physical and mental aspects of their general and disease-specific HRQoL during the course of the study. These improvements were observed starting from the first follow-up survey after three natalizumab infusions and were sustained throughout the study (Figure 2A, and 2b). This translated into a positive outcome for a majority of patients; 79% to 84% of patients reported either a meaningful improvement or no change in their general HRQoL and 85% to 86% of patients reported improved or stable disease-specific HRQoL after 12 infusions (Figure 2C).

### Ability to perform daily activities and disability level

**Figure 3:**
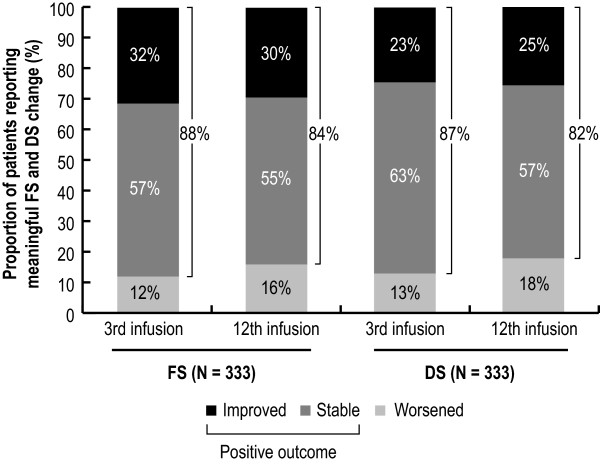
**Changes in ability to carry out daily activities and disability level over 12 natalizumab infusions.** Proportion of patients with improved, no change or worsened ability to carry out daily activities (FS) or disability level (DS) after the 3rd and 12th natalizumab infusion are shown. Score changes corresponding to at least one step in the FS or DS scales were required for the patient’s ability to carry out daily activities or disability level to be considered improved or worsened. *DS* Disease Steps; *FS* Functional Status.

After three natalizumab infusions, 88% and 87% of patients reported a positive outcome in their ability to perform daily activities and their disability level compared with baseline, respectively, seen as either a meaningful improvement or no change in FS or DS score. The effect was sustained to after 12 natalizumab infusions (Figure 3).

### Cognitive functioning and fatigue

**Figure 4:**
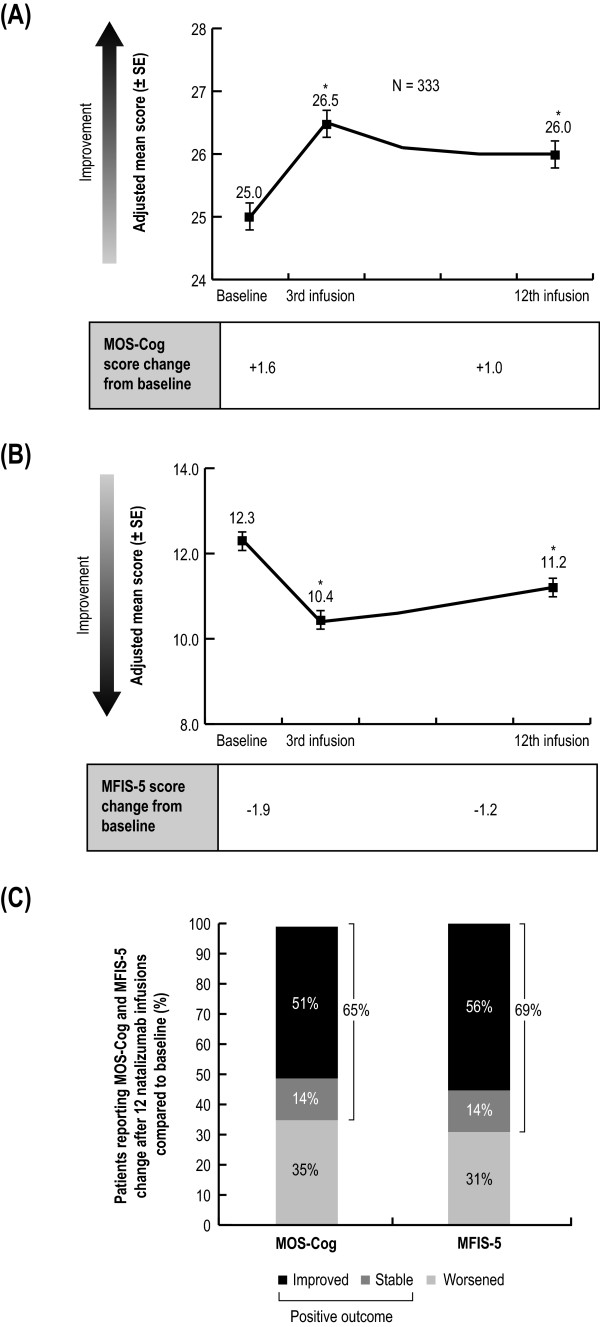
**Changes in the impact of MS on cognitive functioning and fatigue over 12 natalizumab infusions.** (**A**) Mean MOS-Cog (± SE) and (**B**) MFIS-5 scores (± SE) at baseline prior to natalizumab initiation and after the 3rd and 12th infusion. Because of missing data for covariates in their respective models, n values for MOS-Cog were 331 and 333 for MFIS-5. p values were from analyses of variance performed on the adjusted means estimated by the mixed effect model controlling for infusion, age, baseline DS, baseline FS, years since diagnosis, and infusion, age, baseline DS, baseline FS, number of comorbidities for MOS-Cog, and number of MS drugs taken prior to natalizumab for MFIS-5. (**C**) Proportion of patients with improved, stable, or worsened cognitive functioning (MOS-Cog) and fatigue (MFIS-5). MFIS-5 and MOS-Cog scores had to change by at least one score point to be considered changed. *p < .0001. *DS* Disease Steps; *FS* Functional Status; *MFIS* Modified Fatigue Impact Scale - 5 items; *MOS-Cog* Medical Outcomes Scale-Cognitive Functioning; *SE* standard error.

After three natalizumab infusions, patients reported a statistically significant reduction in the impact of MS on cognitive functioning and fatigue compared with baseline, demonstrated by increased MOS-Cog scores and decreased MFIS-5 scores (Figure 4A, 4B). The improvement in cognitive functioning with natalizumab was sustained to after 12 infusions. Compared with baseline, 65% to 69% of patients experienced either a meaningful improvement or no change in the impact of MS on cognition and fatigue after 12 natalizumab infusions (Figure 4C).

### Sensitivity analysis

A comparison of the baseline demographics and characteristics (age, years since MS diagnosis, number of MS drugs prior to natalizumab, number of comorbidities, DS and FS level, proportion of females, proportion of Caucasians, education level, employment status, and income) of the 1,275 patients who completed the baseline survey did not show any significant difference from those of the 333 patients who completed the 12-month survey. A further sensitivity analysis was performed using a mixed effects model with all available patient data at each time point and the same covariates as the linear mixed-effect models, using patient data included in the final analysis. The resulting SF-12v2 (MCS and PCS), MSIS-29 (psychological and physical impact), MOS-Cog, and MFIS-5 scores for the 3- and 12-month time points using this analysis were significantly different from baseline (all p < .0001), which was consistent with the results of the 333 patients who completed the 12-month survey (with the exception of SF-12-v2 PCS; p = .056 at 3 months compared with baseline).

## Discussion

PROs range from subjective measures, such as perceived quality of life or the ability to perform daily activities, to more objective and symptomatic measures, such as levels of physical disability. Some of these aspects are difficult to measure clinically, but nonetheless contribute considerably to the experience of MS. The effectiveness of a therapy in patients’ lives as a whole can therefore be assessed using PRO measures in addition to clinical data. Another factor impacting the effectiveness of a therapy that is difficult to address in a clinical trial is the heterogeneity of a general patient population, since certain patients groups (for example, those with comorbidities) that may confound the results in the trial are excluded. Real-world observational studies, therefore, complement evidence from clinical trials, and evidence of real-world effectiveness is becoming increasingly important in health technology assessments and in the development of treatment guidelines.

MS is associated with a multitude of mental and physical symptoms [1], which, despite being difficult to measure clinically, can be perceived as debilitating by patients. PROs can be used to assess the impact of these symptoms on patients’ lives. Natalizumab is an effective treatment that has been shown to reduce relapses and delay disability progression [8]. This longitudinal study provides evidence that further supports the effectiveness of natalizumab and suggests a positive effect on PRO measures, which were observed from 3 months after treatment initiation and sustained over the 12-month study period.

At the start of the study, most patients reported that MS had a considerable negative impact on their lives, affecting both physical and psychological aspects, despite already having received an average of two DMTs. The patients’ general HRQoL scores were below the scores of the average US population, and the majority had problems with mobility and the ability to carry out daily activities. This poor health status is typical for MS patients, who consistently have a lower HRQoL than both the population without MS [3,27,28] and patients with other chronic diseases, such as diabetes, heart disease, stroke, and arthritis [29]. Fatigue is also a common problem for MS patients; in fact, the majority describe fatigue as the worst or one of the worst symptoms [25]. Fatigue can also exacerbate other symptoms, such as balance, vision, and cognitive problems [29,30].

Because MS is a progressive disease, the absence of a worsening of symptoms is a positive outcome. Following initiation on natalizumab, 65% to 88% of patients reported scores indicating a positive outcome in all PRO measures assessed. What is more, not only was there no further deterioration of the PRO measures assessed, but symptoms were actually improved in a substantial proportion of the patients. Approximately half of patients reporting a positive outcome in their general and disease-specific HRQoL assessments had meaningful improvements in their scores after 12 months compared with baseline. This is consistent with improvements in HRQoL reported by natalizumab-treated patients in randomized clinical trials [11] and in a 3-month real-world study [12]. Similarly, more than a quarter of patients reported improvements in their disability level, their ability to carry out daily activities, and the impact of MS on fatigue and cognition. Improvements such as these have previously been reported in small European studies, in which natalizumab was associated with decreased fatigue [13], a reduction in cognitive impairment [31], and improvements in mobility and reduced disease activity in patients previously treated with interferon beta or glatiramer acetate [32].

Improvements in all PRO measures investigated were already observed after three natalizumab infusions and were sustained over the 12-month study period. This indicates that the impact of natalizumab occurs rapidly. Post hoc analyses from the AFFIRM and SENTINEL clinical trials demonstrated that within the first 3 months of natalizumab treatment, a significant reduction in annualized relapse rate occurred, and that the reduced rate was maintained throughout the 2-year study period. This rapid effect of natalizumab on the annualized relapse rate has also been observed in the TOP study, a clinical practice–based observational study [33].

There are no universally acknowledged minimal score changes that correspond to clinically meaningful changes in patients for any of the PRO measures used in this study, with the exception of SF-12v2 (which, as it is normalized, is directly comparable to the scoring of SF-36, for which a score change of at least 5 constitutes a clinically meaningful change [18]). However, the steps in the FS and DS scales correspond to clearly defined levels of ability or disability (see Additional files 1 and 2) and a change of one step is therefore a clearly noticeable difference to the patient. Similarly, a MSIS-29 score change of at least one category, as defined by the creator of the measure, which corresponds to different degrees of problems caused by MS, would presumably be a meaningful difference for the patient. For both MOS-Cog and MFIS, a 1-point score corresponds to a defined category of how often MS impacts aspects of cognition and fatigue. It is therefore reasonable to assume that the score changes reported are meaningful to the patient.

This was an observational, single-arm prospective study and, as such, has recognized limitations. Since the data presented are longitudinal comparisons within the same population, it is uncertain whether the reported improvements in PROs are a direct result of treatment with natalizumab. However, evidence from randomized clinical trials has shown that, in contrast to natalizumab-treated patients, the HRQoL of MS patients receiving placebo worsens over time [11]. Some of the PRO data could also be affected by recall bias, as the patients were asked to consider a period of up to 4 weeks in the past. In addition, results could be affected by selection bias, as the full set of 12-month data was only available for 333 of the 1,275 patients who were enrolled in the study. However, most of the patients were excluded from analysis because they did not meet study eligibility criteria. In addition, a sensitivity analysis comparing the demographics and characteristics of the patients completing the 12-month survey found that they were not significantly different from those of the patient group completing the baseline survey. Furthermore, a sensitivity analysis that included data for all available time points from all patients who received at least one natalizumab infusion showed similar sustained improvements over time. The self-reported MOS-Cog instrument used to determine cognitive impairment does not provide an objective measure of cognitive functioning similar to that obtained through neuropsychological testing. However, our intent in using the instrument was not to provide a quantitative measure of cognitive functioning but to determine how day-to-day cognitive impairment was perceived by patients and how those perceptions changed over time. In this regard, the MOS-Cog is a reliable scale [24]. Although it is preferable to use the long form of the MFIS in evaluating the impact of fatigue, the abbreviated version, the MFIS-5, was developed for occasions when time is limited, such as in the survey reported here [26]. The MFIS-5 consists of the five items most strongly correlated with the total MFIS score, and the items assess the impact of fatigue in terms of physical, cognitive, and psychosocial functioning. The use of the abbreviated scale thus provided a reliable snapshot of the effects of fatigue given the limited time available.

## Conclusions

Overall, this longitudinal, real-world study demonstrates that natalizumab leads to improvements in PROs from as early as after 3 months of treatment, and that these positive effects are sustained for 12 months. The improvements in PRO measures show that the reported clinical effects of natalizumab are translated into tangible benefits for patients, improving their mental and physical health and positively impacting many aspects of their daily lives.

## Endnotes

^a^Tysabri is a registered trademark of Elan Pharmaceuticals Inc, San Francisco, CA, USA. ^b^TOUCH is a registered trademark of Elan Pharmaceuticals Inc, San Francisco, CA, USA.

## Abbreviations

DMT: Disease modifying treatment; DS: Disease Steps; FS: Functional Status; HRQoL: Health-related quality of life; MCS: Mental Component Summary; MFIS-5: Modified Fatigue Impact Scale 5; MOS-Cog: Medical Outcomes Scale-Cognitive Functioning; MS: Multiple sclerosis; MSIS-29: Multiple Sclerosis Impact Scale 29; PCS: Physical Component Summary; PRO: Patient-reported outcome; SAS: Statistical Analysis System; SF-12v2: Short-Form 12 Version 2; TOUCH: Tysabri Outreach Unified Commitment to Health.

## Competing interests

Judith J. Stephenson is an employee of HealthCore, a research and consulting company. All of her research activities are industry-sponsored. However, she receives no direct compensation as a result of grants or contracts, other than her salary from HealthCore. David M. Kern receives no direct compensation as a result of grants or contracts other than his salary from HealthCore. Sonalee S. Agarwal is an employee of Biogen Idec. Ruth Zeidman is an employee of Medaxial Group, but did not receive any direct compensation for her involvement other than her regular salary. Krithika Rajagopalan was an employee of Biogen Idec at the time of the study. Siddhesh A. Kamat is an employee of HealthCore and receives no direct compensation as a result of grants or contracts other than his salary from HealthCore. John Foley currently sits on several scientific advisory boards supported by Biogen Idec (since 2008), Avanir (since May 2011), and Genzyme/Sanofi (since April 2011). He has been a participant in speakers bureaus for both Biogen Idec and Teva (since 1995), he is a participant in the speakers bureau for Questcor (since July 2011), and he is a consultant for Genzyme, Avanir, Questcor, and Elan. Dr. Foley does not receive stock, stock options, or royalties from any of these entities.

## Authors' contributions

JJS participated in the design of the study, development of the survey and data collection procedures, statistical analyses, interpretation of the data and drafting of the manuscript. DMK helped draft the statistical analysis plan, performed the analysis, and helped draft the manuscript. SSA participated in data collection, analysis, interpretation and drafting of the manuscript. RZ participated in the interpretation of the data and drafting of the manuscript. KR participated in study design, data collection, and manuscript review. SAK participated in development of the survey and the analysis plan, interpretation of the data, and manuscript review. JF assisted with data interpretation and manuscript review. All authors read and approved the final manuscript.

## Supplementary Material

Additional file 1 Functional status (FS) level description. Click here for file

Additional file 2 Disease steps (DS) description. Click here for file
