# Supplementary material for: Impact of natalizumab on patient-reported outcomes in multiple sclerosis: a longitudinal study
Source: Health Qual Life Outcomes. 2012 Dec 27;10:155. doi: 10.1186/1477-7525-10-155 (PMC3543243; doi:10.1186/1477-7525-10-155)
Supplement: Additional file 1 — Functional status (FS) level description. [file 1477-7525-10-155-S1.doc]

**Additional File 1**

Functional status (FS) level description

| **FS level** | **Description** |
| --- | --- |
| 1 | Able to carry out usual daily activities without limitations |
| 2 | Limitations, but can carry out most usual daily activities, even if some require provisions such as altered work hours or naps |
| 3 | Able to carry out about only half of usual daily activities even with special provisions |
| 4 | Severely limited in ability to carry out usual daily activities |
| 5 | Require assistance with even basic self-care activities such as dressing, bathing, transferring, and going to the bathroom |
