# Supplementary material for: Impact of natalizumab on patient-reported outcomes in multiple sclerosis: a longitudinal study
Source: Health Qual Life Outcomes. 2012 Dec 27;10:155. doi: 10.1186/1477-7525-10-155 (PMC3543243; doi:10.1186/1477-7525-10-155)
Supplement: Additional file 2 — Disease steps (DS) description. [file 1477-7525-10-155-S2.doc]

**Additional File 2**

Disease steps (DS) description

| **DS** | **Label** | **Description** |
| --- | --- | --- |
| 0 | Normal | Functionally normal with no limitations on activity or lifestyle |
| 1 | Mild disability | Mild symptoms and/or signs |
| 2 | Moderate disability | Main feature is a visibly abnormal gait |
| 3 | Early cane | Use a cane/unilateral support for walking greater distances, but can walk at least 25 feet (7.6 m) unaided |
| 4 | Late cane | Unable to walk 25 feet unaided without a cane/unilateral support |
| 5 | Bilateral support | Require bilateral support to walk 25 feet |
| 6 | Wheelchair | Essentially confined to a wheelchair |
